# Supplementary material for: Graphene Oxide Strengthens Gelatine through Non-Covalent Interactions with Its Amorphous Region
Source: Molecules. 2024 Jun 6;29(11):2700. doi: 10.3390/molecules29112700 (PMC11173959; doi:10.3390/molecules29112700)
Supplement: Supplementary file 1 [file molecules-29-02700-s001.zip › molecules-3010527-supplementary.pdf]

Supplementary Information

Graphene oxide strengthens gelatine through non-covalent interactions with its amorphous region

Hak Jin Sim [1,2], Katarina Marinkovic [1,2], Ping Xiao [2,3], and Hui Lu [1,*]

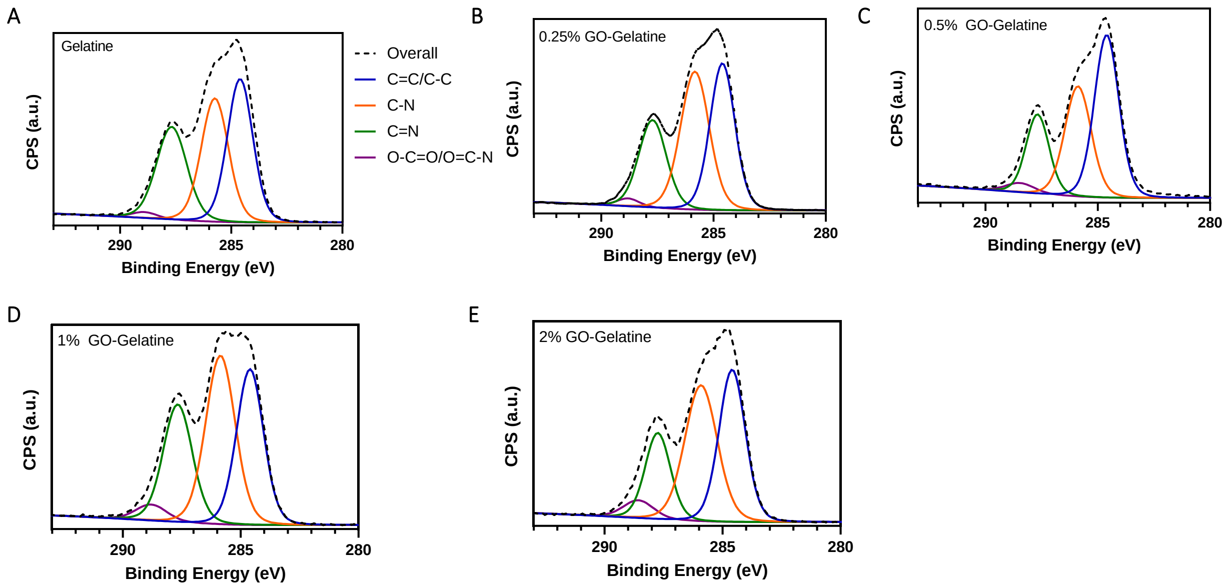

Figure S1. High-resolution XPS C1s deconvolution spectra of gelatine and various GO-gelatine composite samples. (**A**) Gelatine, (**B**) 0.25% GO-gelatine, (**C**) 0.5% GO-gelatine, (**D**) 1% GO-gelatine and (E) 2% GO-gelatine. All the color representations are as indicated in **A**.

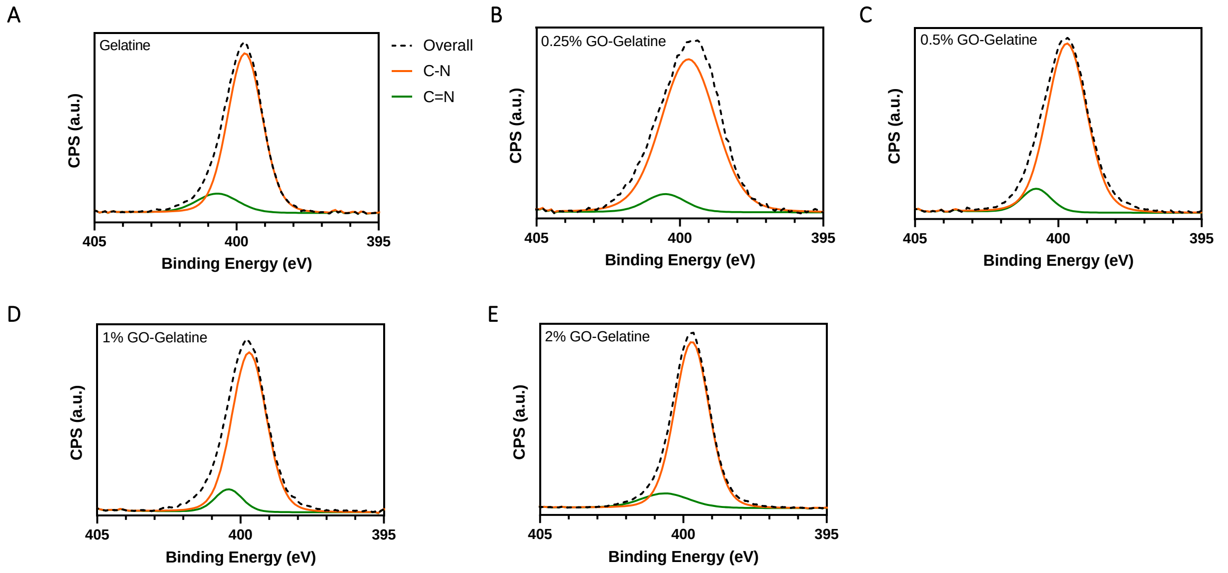

Figure S2: Deconvolution of XPS N1s spectra of gelatine and various GO-gelatine composite sample. (**A**) Gelatine, (**B**) 0.25% GO-gelatine, (**C**) 0.5% GO-gelatine, (**D**) 1% GO-gelatine and (**E**) 2% GO-gelatine. The color representations are as indicated in **A**.
